# Measuring respiratory and heart rate using a fiber optic interferometer: A pilot study in a neonate model

**DOI:** 10.3389/fped.2022.957835

**Published:** 2022-12-05

**Authors:** Jakub Cubík, Stanislav Kepak, Hana Wiedermannova, Adela Vrtkova, Hana Burckova, Pavla Zarubova, Carlos Fernandez, Jan Pavlicek, Jan Jargus, Vladimir Vasinek

**Affiliations:** ^1^ Department of Telecommunications, Faculty of Electrical Engineering and Computer Science, VSB—Technical University of Ostrava, Ostrava, Czech Republic; ^2^ Department of Neonatology, University Hospital Ostrava, Ostrava, Czech Republic; ^3^ Faculty of Medicine, University of Ostrava, Ostrava, Czech Republic; ^4^ Department of Applied Mathematics, Faculty of Electrical Engineering and Computer Science, VSB—Technical University of Ostrava, Ostrava, Czech Republic; ^5^ Department of the Deputy Director for Science, Research, and Education, University Hospital Ostrava, Ostrava, Czech Republic; ^6^ Centre for Cardiovascular Research and Development, American Heart Poland Inc, Kostkowice, Poland; ^7^ Department of Pediatrics and Prenatal Cardiology, University Hospital Ostrava, Ostrava, Czech Republic; ^8^ Biomedical Research Center, University Hospital Hradec Kralove, Hradec Kralove, Czech Republic

**Keywords:** non-contact monitoring, interferometer, biosensor, vital signs, animal testing, newborn

## Abstract

**Introduction:**

The study aim was to test the safety and efficacy of a pad with optic fibers developed for monitoring newborn respiratory rate (RR) and heart rate (HR).

**Methods:**

Thirty New Zealand White rabbits were included, divided by weight into three groups. RR and HR were measured using two methods for each rabbit: ECG electrodes as the reference method and a newly developed pad with an experimental fiber optic system (EFOS) as the experimental method.

**Results:**

Analysis was performed on data for 29 rabbits (10 female, 34%; 19 male, 66%). EFOS performed better at measuring RR compared with HR. RR values did not differ significantly between the methods for the whole group (*p* = 0.151) or within each sex (female: *p* > 0.999; male: *p* = 0.075). Values for HR, however, did differ between methods for the whole group of animals (*p* < 0.001) and also within groups by sex (female: *p* < 0.001; male: *p* = 0.006).

**Conclusion:**

The results of this preclinical study demonstrate the potential of this non-invasive method using a fiber optic pad to measure HR and RR.

## Introduction

Vital signs monitoring is an essential part neonatal clinical care. Monitored parameters include heart rate (HR), respiratory rate (RR), and peripheral blood oxygen saturation (1). Monitoring methods are classified as invasive vs. non-invasive or as contactless vs. contact. Non-invasive methods are more common and rely on electrodes or sensors attached to the skin, with standard monitoring devices. The gold standard for HR is electrocardiography (ECG), and RR is most often measured using chest impedance from signals obtained from ECG electrodes (2).

Despite their non-invasive nature, these methods can have side effects. With contact methods, monitoring causes stress to the newborn, especially immature newborns, and increased infection risk *via* skin damage when electrodes are changed (3). Another disadvantage is the need to handle the newborn, negatively affecting circadian rhythm and sleep patterns, along with disruptions from alarms, device cables, the sound of repeated opening or closing of the incubator, and olfactory sensations when disinfectant is used. For these reasons, research into vital signs monitoring focuses on non-invasive, non-contact technologies, such as video monitoring, Doppler ultrasound, and infrared cameras (4). The current main non-contact method is an apnea pad with an internal piezoelectric pressure sensor, placed under the mattress. An absence of breathing movements triggers an alarm.

The aim of this preclinical study was to test the safety and efficacy of a newly developed optical fiber–based pad monitor of RR and HR. Here, we present results in an animal model intended to simulate newborn size and physiology.

## Materials and methods

### Animals and experimental design

Because of potential similarities to human neonates, New Zealand White rabbits were used in this investigation, based on recommendations from experts in preclinical studies (5). Weight and vital sign parameters (HR, RR) in this animal correspond closely to those of human neonates. Normal HR in these rabbits is 130–325 beats/min, and RR is 30–60 breaths/min (6).

A total of 30 rabbits were included, divided by weight into three groups. The first group consisted of six animals (two female, four male) weighing 1.5–1.9 kg, the second group consisted of 13 (five female, eight male) weighing 2.0–2.9 kg, and the third group consisted of 11 rabbits (four female, seven male) weighing >3.0 kg. Each animal was healthy, with an associated card noting the animal strain, sex, weight, date of arrival at the research center, and a unique identification number.

RR and HR were measured in each rabbit in two ways. The first, standard method (reference) involved monitoring HR and RR using ECG electrodes (Philips IntelliVue MP5 Pediatrics monitor). Each rabbit was monitored in the supine position, with the chest shaved and standard ECG electrodes designed for neonatal use glued on. Monitoring was performed for 5 min.

The second method was experimental, involving the special pad with optical fibers. Each rabbit was placed on its back on the pad, and monitoring was carried out for 3 min, while the animal also was monitored with ECG electrodes.

### Description of the experimental system

**Figure 1:**
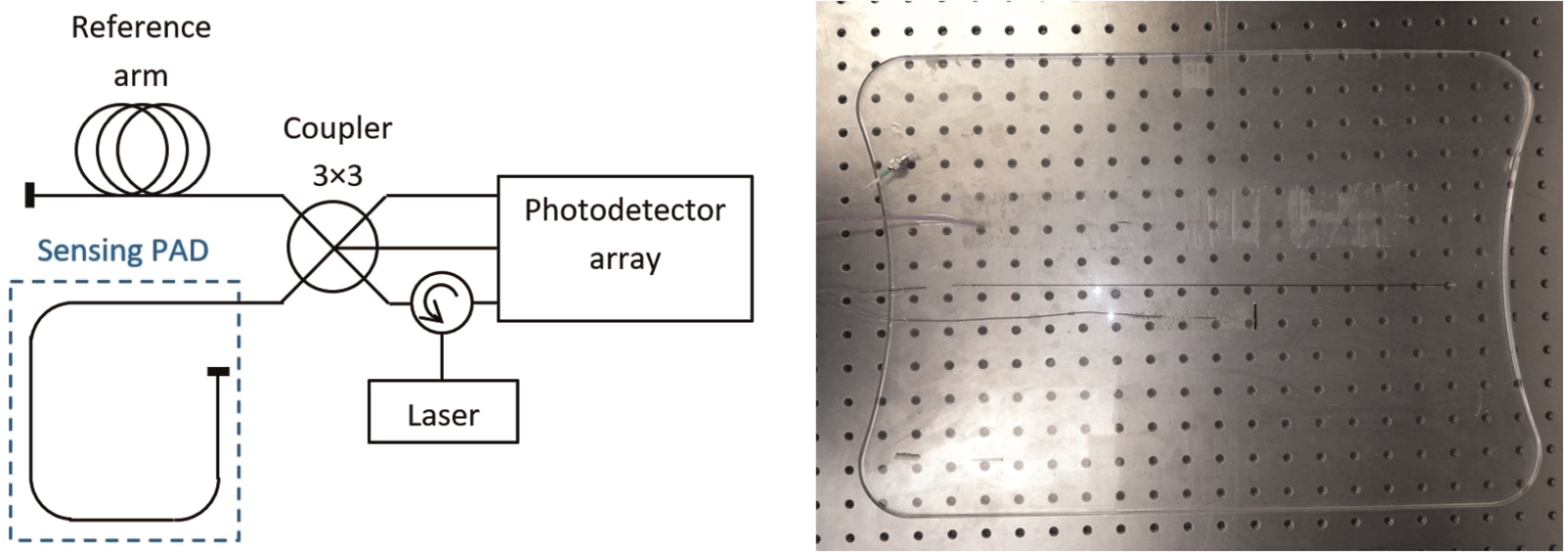
Schematic diagram of the experimental sensor and sensing pad.

The experimental optic fiber system (EFOS) used here comprises a sensing pad with an embedded optical fiber in primary protection, terminated with a mirror. In an optical fiber, changes in external conditions (e.g., vibration) change the optical path length (OPL) and thus the phase delay of the light passing through the fiber. The sensing pad was made in several steps. First, a mold for pouring the elastomer was selected, and the first layer (0.3 cm) of elastomer was then poured. The optical fiber was placed on the first layer so that the edges of the structure were 3.5 cm from the edge of the mold. The fiber structure was held in the axis of the mold. After the fiber structure was centered, it was locally fixed with an elastomer and covered with a second layer (0.3 cm) to produce a pad measuring 50 cm × 34 cm and weighing 1.3 kg. Sylgard 184 elastomer was used for encapsulation (Figure 1).

This part forms the measuring arm of the fiber optic interferometer, which further consists of a reference arm with a similar OPL and a fiber coupler. The difference in OPLs between the reference and measuring arms was converted by the interferometer into a modulated form according to the following formula:

I(t)=C+Acos⁡[OPLD(t)],

where C is the mean value of the resulting optical intensity, A is the amplitude of the variation of the optical intensity, and OPLD is the optical path length difference between the measurement and the reference path. It was essential to apply a demodulation technique to unwrap the phase shift and acquire the sensor data. The method of passive homodyne demodulation described in (7, 8) was used to demodulate the sampled signal, so that a coupler was used that had three input and three output ports (3 × 3) with a uniform split ratio, with outputs that could be characterized by the following relation:

$$u_{k}\left(t\right)=C_{k}+A_{k}\;\cos\left[\mathrm{OPLD}\left(t\right)+\left(\frac{2\pi }{3}\right)\cdot k+\delta_{k}\left(t\right)\right],$$

where k = 1,2,3 and *δ_n_* is 3 × 3 coupler phase asymmetry. Before the demodulation, all three channels are equalized so that *C_k_*_ _= 0 and *A_k_*_ _= 1. The OPLD could then be unwrapped using the following equation combining the three output signals from the optical coupler:

$$OPLD\left(t\right)=\arctan\left\{\frac{\sqrt{3}\left[u_{2}\left(t\right)-u_{3}\left(t\right)\right]}{u_{2}\left(t\right)+u_{3}\left(t\right)-2u_{1}\left(t\right)}\right\}.$$

An optical fiber circulator was used to connect the radiation source, which was a laser diode operating at 1540 nm with a power of up to 10 mW. The photodetector array consisted of Roithner PD-1375-IP photodiodes and a variable bandwidth up to 2 MHz.

**Figure 2:**
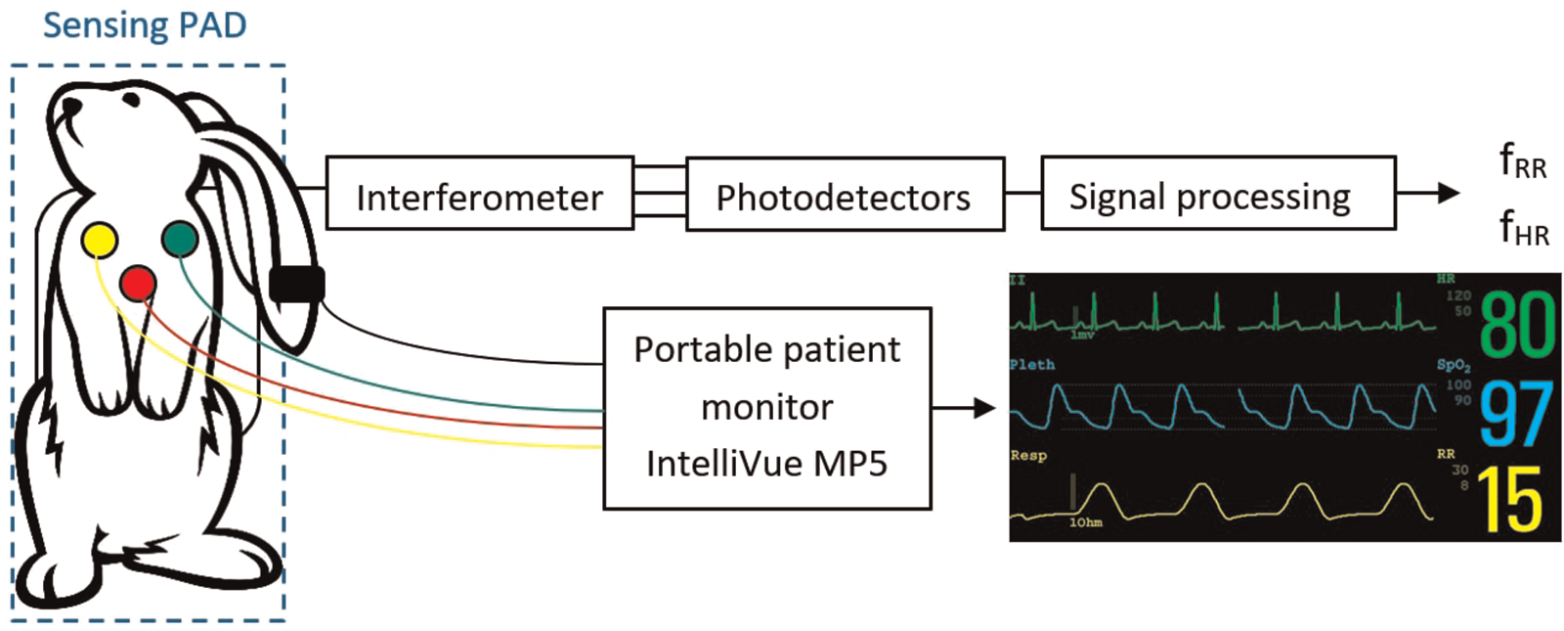
Measurement setup.

Sampling was performed using a National Instruments 9,239 module with a sampling rate of up to 50 kS/s/ch housed in a cDAQ-9171 chassis. This analog-to-digital converter is supported in the LabVIEW development suite, which was used to develop a custom application for data logging and subsequent signal processing. The basic measurement scheme is shown in Figure 2.

### Procedure description and subsequent signal processing

HR and RR measurements were recorded at 1-min intervals. ECG monitoring was performed for 5 min, followed by EFOS monitoring for 3 min, while ECG monitoring continued simultaneously. Thus, for each rabbit, three pairs of HR and RR measurements (one value from the ECG electrodes, the other value at the same time point from EFOS, values obtained during 5 min of simultaneous measurement) were initially available to assess the reliability of EFOS.

**Figure 3:**
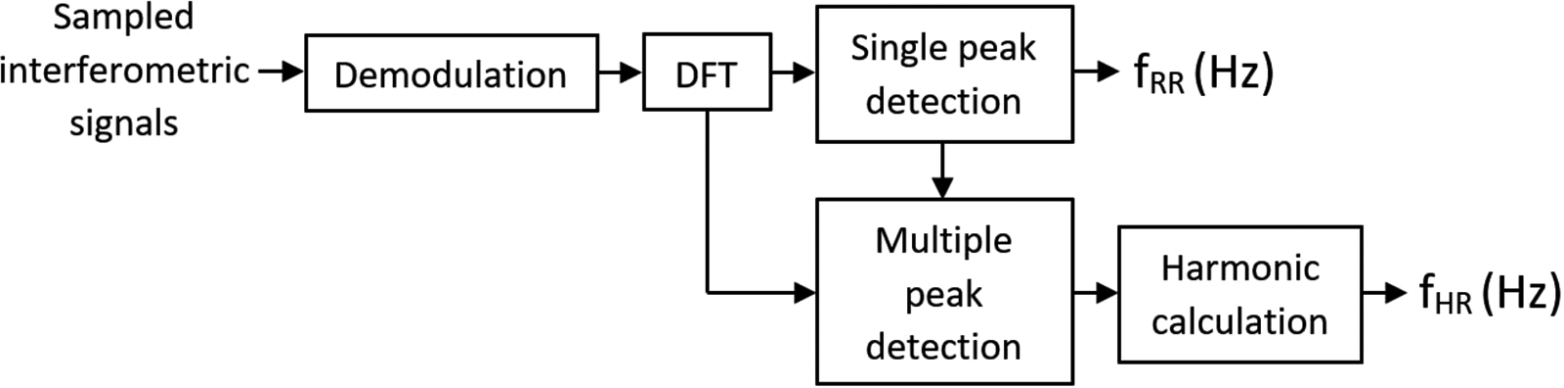
Block diagram of signal processing.

Signal processing was performed as shown in Figure 3. The sampled output signals from the interferometer were demodulated at first. This process can provide information about the phase change measured by the interferometer. A passive homodyne demodulation technique was used for demodulation, as discussed in detail in (6). A discrete Fourier transform was performed on the signal, yielding its frequency spectrum. The parameters for the Fourier transform algorithm were a window length of 60 s (30 s in case of distortion, e.g., by spontaneous movement of a rabbit), and the Hamming window function was applied.

According to the reference method (ECG), our rabbits had a RR range of 50–133 breaths/min and HR range of 101–224 beats/min. Clearly, a wholly consistent association cannot be drawn between RR and HR, but it is always true that RR < HR. Thus, the algorithm for the calculation first identified the maximum possible value of amplitude in the frequency range >0.5 Hz, with the upper cutoff frequency, based on observations, capped at 2.5 Hz, so that this maximum value then always corresponded to the RR.

**Figure 4:**
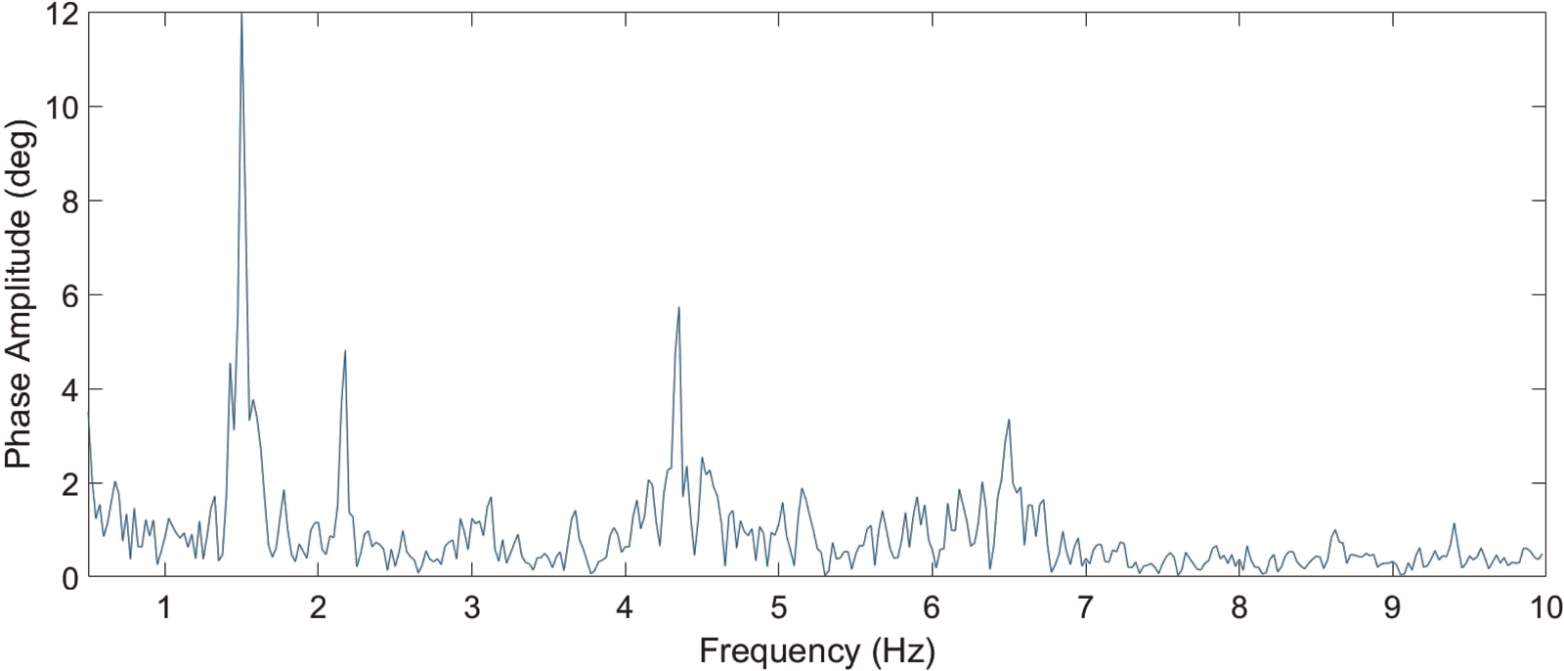
Sample of measured spectrum and measured frequencies for RR and HR.

In the next step, the HR was searched within the five maxima in the frequency range starting at 1.5 Hz (if the RR was greater than or equal to this value, RR + 0.25 Hz was used). Of these maxima, the lowest value from the expected range of valid frequencies that had at least one additional harmonic component was used as the HR. A sample measured spectrum is given in Figure 4.

Motion artefacts occurred during measurements, which prevented automatic determination of vital signs at given time intervals. For this reason, the entire record of one rabbit also was excluded from further processing. A total of 81 valid HR measurements (90.0% of the total number of measurements) and 78 valid RR measurements (86.7% of the total) remained for the other rabbits (*n* = 29), excluding records containing these artifacts.

This portion of study involving animals was reviewed and approved by the Ethics Committee for Animal Experiments in Katowice, Medical University of Silesia, Poland, No. 96/2019.

### Statistical analysis

Statistical analysis was performed with R software (version 4.1.1, www.r-project.org). The significance level was set to 0.05. Agreement between methods was analyzed with correlograms and Bland–Altman plots with non-parametric limits of agreement (2.5% quantile and 97.5% quantile) and evaluated with intraclass correlation coefficients (ICCs) with corresponding confidence intervals. Differences between methods are described with medians and interquartile ranges (IQRs, i.e., lower and upper quartiles), analyzed with the paired Wilcoxon test. The Mann–Whitney *U* test and Levene's test were used to evaluate differences between female and male rabbits. Spearman's rank correlation coefficient (Spearman's rho) was used to assess a relationship between the precision of the EFOS and weight.

## Results

The analysis was performed on data for 29 rabbits (10 female, 34%; 19 male, 66%). Their weights ranged from 1.50 kg to 4.15 kg, with a median of 2.90 kg. Weight did not differ significantly between female and male rabbits (Mann–Whitney *U* test, *p* = 0.854).

The range for HR (as measured by the reference method with simultaneous measuring by the EFOS) was 122 to 202 beats/min, with a median of 158 beats/min. Female rabbits had significantly higher HR compared with males (Mann–Whitney *U* test, *p* = 0.025; median 163 beats/min for females vs. 155 beats/min for males). The variability of the measured HR did not differ between female and male rabbits (Levene's test, *p* = 0.306).

The RR (measured using the reference method during simultaneous EFOS measurement) ranged from 39 to 129 breaths/min, with a median of 77 breaths/min. Female rabbits also had significantly higher RR (Mann–Whitney *U* test, *p* = 0.013; median 88 breaths/min for females vs. 71 breaths/min for males). The variability of the measured RR values did not differ between female and male rabbits (Levene's test, *p* = 0.798).

**Figure 5:**
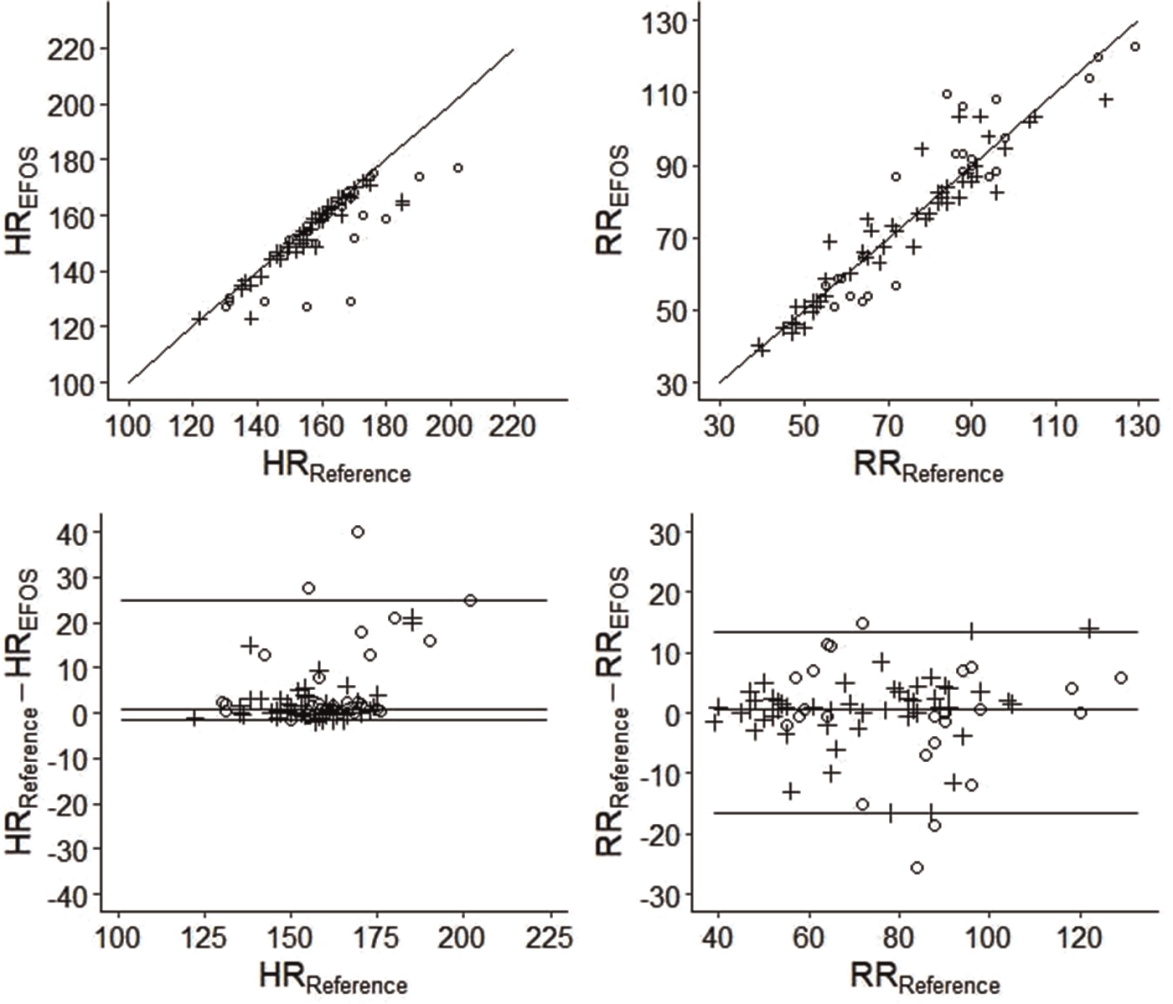
The correlograms (top) and the bland–altman plots (bottom) visualizing the analysis of agreement between the reference and EFOS methods (circles = females; crosses = males).

Analysis of the overall agreement of the reference and EFOS methods is visualized in Figure 5, and a detailed analysis is given in Table 1.

**Table 1:** Analysis of differences between the reference and EFOS methods and evaluation of agreement for all rabbits and for females and males separately.

	Difference (Reference—EFOS)		
	Median (IQR)	*p* *	ICC (95%CI)
**Heart rate (beats/min)**			
Total	1.0 (0.0; 3.0)	<0.001	0.83 (0.69; 0.90)
Female	2.0 (0.5; 13.0)	<0.001	0.72 (0.34; 0.88)
Male	0.0 (−0.5; 2.5)	0.006	0.92 (0.86; 0.96)
**Respiratory rate (breaths/min)**			
Total	0.8 (−1.5; 3.5)	0.151	0.94 (0.91; 0.96)
Female	0.0 (−2.0; 6.0)	>0.999	0.91 (0.81; 0.96)
Male	1.0 (−0.5; 2.5)	0.075	0.96 (0.93; 0.97)

IQR, interquartile range; ICC, intraclass correlation coefficient; CI, confidence interval.

* *P* value of the paired Wilcoxon test.

The results indicate that the EFOS method performed better at measuring RR compared with HR. No significant difference in RR was found between the methods for the group as a whole (*p* = 0.151) or within sexes (females: *p* > 0.999; males: *p* = 0.075). The ICC estimates were >0.9, suggesting strong inter-method agreement (ICCs: all rabbits, 0.94; females, 0.91; males, 0.96). Moreover, there was no significant relationship between sex and differences in measured RR (Mann–Whitney *U* test, *p* = 0.607). As Figure 5 shows, the differences were distributed relatively evenly around 0.

In contrast, a significant difference in measured HR was found between methods for the whole group (*p* < 0.001) and within sex groups (females: *p* < 0.001; males: *p* = 0.006). Significantly higher differences between methods were observed in female rabbits (Mann–Whitney *U* test, *p* = 0.003). However, in females and in males, EFOS rather underestimated HR. The ICC estimate supported this worse performance of EFOS in measuring HR; the ICC values were still relatively high (>0.7) but lower than when measuring the RR and with wider confidence intervals.

Moreover, the analysis showed a significant negative correlation of weight with differences between methods for RR (Spearman's rho: −0.23, 95% confidence interval [CI]: [−0.43, −0.01], *p* = 0.044). This result suggests that with increasing weight, EFOS measurements for RR became more precise. Additionally, weight and differences between methods for HR were not significantly correlated [Spearman's rho: −0.04, 95% CI: (−0.26, −0.18), *p* = 0.711].

## Discussion

Non-contact monitoring of newborns is an integral part of hospital care, and also is important and has a stable place in home care. Monitoring helps to prevent sudden infant death syndrome and is recommended especially for at-risk newborns. Non-contact monitoring has undeniable advantages, including eliminating stress and discomfort for the baby. There is no skin damage from application of electrodes or sensors, reducing infection risk and eliminating negative sensory perception. The most commonly used pads for monitoring breathing activity rely on pressure sensors placed in the pad (2, 4). Other non-contact methods are being developed for greater advantages. The key question is whether these methods can accurately measure heart and breathing rates (9, 10).

Here we focused on a non-contact fiber optic sensing pad, seeking to verify its applicability in a preclinical model. The pad is placed underneath the subject or under a mattress and requires no other sensing equipment. The stress or pain of the measurement is minimized, and the risk of skin damage or infection is eliminated. The result is numerical values for breathing and heart rate, which this device can measure simultaneously. We have compared this methodology with other non-contact methods focusing on respiratory and heart rate (9, 10).

Currently developed non-contact methods for monitoring breathing include remote photoplethysmography, infrared thermal imaging, or RGB (red, green, blue) camera, techniques based on the Doppler principle (4, 9, 11–13). Most of these methods are accurate but require a transmitting (source) and sensing (detector) device to be placed near the patient. Limitations include patient location, device size, and cost. Options for passive sensing of RR include capacitive sensors or piezoelectric and piezoceramic sensors (14, 15), placed in the crib under a sheet or mattress, similar to our pad. The apnea monitor is an example of a piezoelectric sensor placed in the pad, and the most commonly used, but these monitors assess breathing rate only by presence or absence, do not generate numerical values, and do not assess heart action at all. A more accurate mat is an ultrathin foil with pressure sensors (15) that also is placed under the mattress. Results for RR, compared with values from thoracic impedance, are very good with this method. Our developed pad is similar in design to the apnea pads: They do not require an additional sensing device, they can give numerical values for RR, and they show good correlation with the reference method. No significant difference in RR measurement was found between the methods, and they showed strong agreement between them. Our results show very good performance of the EFOS in measuring the RR.

Regarding HR measurements, non-contact methods include those used for RR (10, 11), with other possibilities such as dynamic light scattering (16). There is a high correlation with the cardiac action measured by ECG electrodes, but the same limitations as for RR apply with these methods for HR. The HR measurement is possible with piezoelectric (pressure) sensors placed under the mattress cover in a cot or incubator (14). The fiber optic pad evaluated in the current work can sense HR simultaneously with RR, but the agreement between this method and the reference method was not as good as for RR. The differences between them were significant, and EFOS tended to underestimate real HR as measured with the reference method. One possible explanation for this weaker performance is minor motion artifacts, which can lead to the loss of one period of the EFOS signal. Higher differences between methods in HR were observed in female rabbits, possibly because of their generally higher HR compared with males. Another reason EFOS seems to have performed better with RR could be that breathing is generally a stronger phenomenon than a heartbeat and thus easier to measure with the EFOS.

The aim of this study was to verify whether the pad evaluated here can measure RR and HR. We deliberately chose an animal model with a weight of 1500 g or more, because children under this weight are typically monitored with pulse oximetry, a contact method. The results have proved favorable. The advantage of our pad is certainly that it allows adherence to the non-contact principle of measurement with all of its advantages, does not require additional add-on devices, and likely would have a favorable production cost. This study was a preclinical evaluation using animals that had to be put to sleep, so how the pad would perform with neonates in motion remains to be tested.

## Conclusion

The results of this study using an animal model demonstrate the potential of a non-invasive fiber optic pad for measuring HR and RR. The pad produced good measurements without the need for adhesive electrodes and risk for skin damage. It demonstrated effectiveness in the measurement of RR but gave HR values that showed significantly greater variation compared with measurements using a reference method. The pad-based method is only in the early stages of testing and development, and its development is expected to continue in a planned study with newborns.

## Additional requirements

The study was conducted at the Center for Cardiovascular Research and Development, American Heart of Poland, Kostkowice, Poland.

## Data Availability

The raw data supporting the conclusions of this article will be made available by the authors, without undue reservation.
